# Soil Microbial Network Complexity Varies With pH as a Continuum, Not a Threshold, Across the North China Plain

**DOI:** 10.3389/fmicb.2022.895687

**Published:** 2022-06-06

**Authors:** Ying Yang, Yu Shi, Jie Fang, Haiyan Chu, Jonathan M. Adams

**Affiliations:** ^1^School of Geography and Ocean Science, Nanjing University, Nanjing, China; ^2^State Key Laboratory of Crop Stress Adaptation and Improvement, School of Life Sciences, Henan University, Kaifeng, China; ^3^State Key Laboratory of Soil and Sustainable Agriculture, Institute of Soil Science, Chinese Academy of Sciences, Nanjing, China

**Keywords:** bacteria, fungi, co-occurrence network, biogeographic model, keystone taxa, soil pH

## Abstract

There has been little study on the biogeographical patterns of microbial co-occurrence, especially in agricultural soils. Here we investigated the biogeographical patterns and major drivers of co-occurrence network topological structure, and the relative abundance of keystone taxa for soil bacterial and fungal communities using high-throughput sequencing on a set of 90 samples across a 1,092 km transect in wheat fields of the North China Plain (NCP). We found that pH was the most important environmental factor driving network topology and relative abundance of keystone taxa. For the metacommunity composed of both bacteria and fungi, and for the bacterial community alone, lower soil pH was associated with a more complex microbial network. However, the network for fungi showed no strong trend with soil pH. In addition, keystone taxa abundance was positively correlated with ecosystem function and stability, and best explained by pH. Our results present new perspectives on impacts of pH on soil microbial network structure across large scales in agricultural environments. This improved knowledge of community processes provides a step toward understanding of functioning and stability of agricultural ecosystems.

## Introduction

Soil microorganisms do not exist as individuals in nature, but are connected as complex ecological webs through associations such as mutualism, competition, parasitism or neutral interactions, which affect how they adapt to environmental fluctuation and change (Faust and Raes, 2012). In order to better understand these complex associations, correlation-based network inference methods have been developed to predict potential microbial associations from large volumes of high-throughput sequencing data (Zhou et al., 2011; Faust and Raes, 2012). In co-occurrence networks, nodes represent individual microbial taxa derived from operational taxonomic units (OTUs), and positive/negative links between nodes represent potential associations between species (Brian and Fath, 2019). Despite the lack of empirical evidence for the ability of network analysis to provide explicable results, there is little doubt that it may help reveal information about community structure and underlying function that is not available from alpha or beta diversity analyses (Fuhrman and Steele, 2008; Weiss et al., 2016), adding a substantial dimension to reveal the stability and complexity of ecological processes and ecosystem functions (Deng et al., 2012). Firstly, network analysis may reveal niche overlap of microorganisms in the community, because taxa that have the similar niche requirements and responses to environmental change may tend to coexist and vice versa (Wan et al., 2020). Secondly, based on network topological features it is possible to identify putative keystone taxa (those that are highly correlated with other taxa in the network), which drive community composition and function irrespective of their abundance (Berry and Widder, 2014; Banerjee et al., 2018). In addition, the network structure quantified by the network topological features—such as network connectivity and stability—will change with environmental disturbance. For example, Yuan et al. (2021) reported that network interactions among different phylogenetic populations in soil microbial communities would change significantly due to climate warming. However, further research is needed to determine whether similar trends occur along other environmental gradients.

There are various mechanisms which may cause phylotypes within the microbial community to co-occur more often than by chance. One is similarity of niches, without direct interdependence. In terrestrial ecosystems, phylotypes belonging to diverse taxa may have similar phenotypic characteristics or life-history strategies (Fierer et al., 2007; Barberán et al., 2017), so that they tend to co-occur. Members of such microbial communities that share niche spaces are identified as ecological clusters (Singh et al., 2010). By identifying dominant phylotypes that have a strong preference for a given environmental condition (e.g., low or high pH), it is possible to use this information to predict their distributions and enhance the ability to actively manage soil communities (Delgado-Baquerizo et al., 2018). For example, Delgado-Baquerizo et al. (2018) classified microbial communities into different ecological clusters based on the association between bacterial abundance and soil environmental characteristics, which verified the possibility of predicting bacterial distribution on a global scale. However, it is not clear whether soil microbes with similar environmental preferences tend to co-occur in farmland ecosystems.

Moreover, relating to co-occurrence patterns is the attribute of microbial niche breadth. This is a comprehensive index reflecting the resources available to species (Levins, 1968). Species with large niche breadth that have strong ability and competitiveness in resource utilization, and are called generalized species or widespread species. Species with narrow niche breadths, on the other hand, are called specialized species (Dolédec et al., 2000). Species with broad environmental niches will be expected to experience fewer specialized and predictable interactions within a co-occurrence network. Thus, the intensity of interactions in the network is likely to be inversely related to how broad microbial niches are, on average. However, this niche width attribute has not been widely explored in the literature on co-occurrence networks.

Exploring the geographic patterns of soil microbial communities can help to discern spatial distribution characteristics more clearly, and provide insights for understanding the spatial aggregation of microbial communities at a range of spatial scales (Ma et al., 2016). Jiao et al. (2019) constructed an atlas of soil bacterial communities in maize and rice fields in eastern China, finding that taxonomic richness in maize fields was higher at high latitudes than that in low latitudes, however, this trend was not found in rice soils. Similarly, Shi et al. (2019) used a species distribution model to predict the soil bacterial diversity and community composition across the North China Plain. However, only a few studies have explored the geographical distribution patterns of the co-occurrence network topological features for microbial communities (Ma et al., 2016; Wan et al., 2020; Chen et al., 2021), focusing on isolated soil bacterial, fungal, and archaea communities. Yet, understanding of the integrated biogeographic patterns of different communities and their underlying drivers remains limited, which is crucial to improving their ecological role and exploring the mechanisms that constitute and sustain ecosystem functions.

In the present work, we sought to increase understanding of broad scale trends in soil microbial community network structure in an important agricultural environment. Our study area was the North China Plain, the most important grain producing area in China (Jeong et al., 2014). In order to comprehensively evaluate the geographic patterns of microbial co-occurrence networks, we used 16S rDNA high-throughput sequencing technology and network analysis to investigate the soils of 90 typical wheat field soil samples. We aimed to address the following questions: (1) What is the biogeographic pattern of soil bacterial and fungal community co-occurrence network in wheat fields of the North China Plain? (2) What ecological factors drive the geographic patterns of network topological features and the connections between kingdoms (bacteria and fungi)? (3) What are the keystone taxa in the community and what are the environmental factors that regulate their relative abundance? Our findings provide a basis for further understanding the biogeographic pattern and environmental impact mechanism of soil bacterial and fungal communities in wheat fields of North China Plain, and may ultimately contribute to prediction and management the microbial communities in farmland ecosystems.

## Materials and Methods

### Soil Sampling and Data Collection

The sampling area of this study extends about 1,092 km from approximately 30°N to 40°N and 109°E to 122°E across the North China Plain, with an average annual temperature varying between 8 and 15°C, and the average annual precipitation varies between 500 and 1,000 mm. This is the most important grain producing region of China, mostly planted with wheat and maize in a rotation, and supported by irrigation, fertilization and high yielding crop varieties (Chen et al., 2004). The soils from the sampling sites were classified as Ochric Aquic Cambosols (within the Chinese soil taxonomy) in this area (Zhu et al., 2005). A total of 90 samples were collected from 24 sites in North China Plain during the winter season (the 20th–30th of November 2014) (Supplementary Figure 1A), when winter wheat was at the tillering stage and fertilizer was not yet applied to the field, which effectively avoided the strong effect of fertilizer on microbial community. In order to ensure the integrity and uniformity of soil pH gradient, we sampled a varying number of plots (each plot is 100 m × 100 m) for each site (within 100 km) (Supplementary Figure 1B and Supplementary Table 1), and collected 12 cores within each plot at a depth of 0–15 cm and mixed as one sample. All the samples were stored in ice boxes and brought back to the laboratory within several hours to minimize temperature changes on the way before they could be stored in the freezer at −20°C. Soil samples were sieved using a 2 mm mesh to remove roots and stones, homogenized in the laboratory.

For each sample, a total of 25 edaphic factors were tested, while only those with Spearman correlation coefficient less than 0.6 were retained for subsequent analysis (Supplementary Figure 2). Finally, environmental factors include soil pH, SM (soil moisture), OC (organic carbon), DOC (dissolved organic carbon), DON (dissolved organic nitrogen), NH_4_^+^ (ammonium), NO_3_^–^ (nitrate), TP (total phosphorous), TK (total potassium), AP (available phosphorous), AK (available potassium), EC (electrical conductivity), K (potassium), Cr (chromium), Mn (manganese), Cu (copper), Zn (zinc), Cd (cadmium), Pb (plumbum), and As (arsenic). Specific measurement methods for each factor have been presented in detail in the earlier published article (Shi et al., 2018). The regional map of North China Plain was obtained from the Resource and Environment Science and Data Center^1^.

### DNA Extraction From Soil and High Throughput Sequencing

DNA was extracted from 0.5 g soil using a MoBio Power Soil DNA extraction kit (MoBio Laboratories, Carlsbad, CA, United States) by following the manufacturer’s instructions, purified with an Ultra Clean 15 DNA purification kit (MO BIO), and stored at −20°C. Bacterial community analysis was carried out using a 16s rRNA genes primer pairs 515F (5′-GTGCCAGCMGC CGCGGTAA-3′)/907R (5′-CCGTCAATTCCTTTGAGT TT-3′) for the V4 hypervariable regions (Biddle et al., 2008), the fungal ITS2 region was amplified by primer sets ITS3 (5′-GCATCGATGAAGAACGCAGC-3′)/ITS4 (5′-TCCTCCGCTTATTGATATGC-3′) (Gade et al., 2013). DNA concentration was measured on a Nano-Drop ND-1000 spectrophotometer (Thermo Scientific, United States). After sequencing, sequences were analyzed using the QIIME pipeline^2^ (Caporaso et al., 2010). The low-quality sequences that had a quality score < 20, contained ambiguous nucleotides, or did not match the primer and barcode, were removed. Operational taxonomic units (OTUs) were generated at 97% similarity cutoff using the UCLUST method in QIIME (Edgar, 2010). Using Greengenes database^3^ to annotate taxonomic information for each bacterial sequence, UNITE database (Kõljalg et al., 2005) to identify fungal taxonomy.

### Statistical Analyses

Meta-community co-occurrence network was constructed using the “WGCNA” package (Langfelder and Horvath, 2012). All core bacterial and fungal taxa in the top 20% relative abundance and presented in more than 60% of all soil samples were included in the network analysis, while those taxa having no robust correlation relationships (Spearman’s correlation coefficients of > 0.6 and false-discovery-rate-corrected *P* values of < 0.01) with other taxa were lost during the generation of the networks. Sub-networks for each soil sample were also generated by subgraph function in the “igraph” package in R (Csardi and Nepusz, 2006). These network images were visualized with Gephi^4^ (Bastian et al., 2009). To describe the topology structure of the network, we calculated a series of topological features (Supplementary Table 2). Node-level topological features describe the ecological location information of each node in the network, nodes with higher node-level topological feature values occupy the core position in the network, whereas lower value nodes are in the peripheral position (Ma et al., 2016). Network-level topological features with a high value (such as number of edges, average degree, clustering coefficient and density) indicate a more complex and connected network, whereas those with lower values (such as average path length and modularity) suggest closer connections and more concentrated within the network (Barberán et al., 2012; Ma et al., 2016).

To test the significance and importance of the environmental variables for network-level topological features, first, multiple regression model (MRM) on Euclidean distance matrices with the R “ecodist” package (Martiny et al., 2011) were used to assess the relative contribution of each non-collinear edaphic variable in shaping the change pattern of overall network-level topological features. Then, kriging interpolation maps were performed in ArcGIS 10.4^5^ to estimate the geographic patterns of the co-occurrence network-level topological features. Pearson correlation coefficients and *P* values of the predicted values of topological attributes and observed values at the point calculated by cor.test function in “stats” package in R (Field et al., 2012) were used as the result of cross validation (“CV”), and a simple linear regression between pH and the network level topological characteristic was shown in each map. Additionally, to test the potential roles of soil pH in bacterial and fungal community assemblages, we clustered the core taxa into two ecological preference groups (High pH and Low pH) based on the significant Spearman correlations (*P* < 0.05) between OTUs abundance and environmental factors (Chen et al., 2021). Random forest analysis was performed to identify the major environmental factors contributing to the relative abundance of dominant taxa in high-pH and low-pH cluster for soil microbiota. The analysis was performed using the randomForest function in the “randomForest” package in R (Liaw and Wiener, 2002).

In order to further illustrate the effect of pH on the network structure, we identified the ratio of link to node as network complexity and compared the network stability of high and low pH clusters. A regression analysis between network complexity and pH based on Pearson correlation was further conducted. Robustness, as a measure of network stability, was calculated by removing nodes in the static network to estimate how easily robustness degraded. Edge information of the high and low clusters pH were obtained and then nodes were randomly and repetitively removed, and natural connectivity of the nodes was used to assess the network robustness (Peng and Wu, 2016). Moreover, to quantify habitat specialization for high and low pH clusters communities, Levins’ niche breadth (B) index was calculated using the formula:

$$B_{i}=\frac{1}{\sum_{i=1}^{N}p_{i\,j}^{2}}$$

where *Bi* represents the habitat niche breadth of species *i*; *N* represents the total number of samples; *Pij* is the proportion of species *i* in sample *j* (Levins, 1968; Pandit et al., 2009; Wu et al., 2018). The habitat niche breadth was calculated using the “niche.width” function in “spaa” package of R. Boxplot were used to illustrate the habitat niche breadth of high and low pH clusters, with *t*-test were used to reveal the significant difference between them.

The within-module connectivity (Zi) and among-module connectivity (Pi) of each node are calculated based on Markov clustering algorithm with the “rJava” package in R to reflect the topological role of each node, the Zi reflects how close a node is connected to other nodes within its own module, and Pi describes how close a node contacts with different modules. The topological roles of different nodes can be categorized into four types: peripherals (Zi ≤ 2.5, Pi ≤ 0.62), connectors (Zi ≤ 2.5, Pi > 0.62), module hubs (Zi > 2.5, Pi ≤ 0.62) and network hubs (Zi > 2.5, Pi > 0.62) (Deng et al., 2012; Cong et al., 2015). Generally, connectors, module hubs and network hubs are considered as putative keystone taxa of ecological network (Olesen et al., 2006). These relatively rare but highly connected taxa play an important role in improving soil functional potential in agricultural ecosystems (Shi et al., 2020). Spearman correlation and multiple regression model were performed to explore the determinants of the identified keystone taxa.

## Results

### An Overview of the Constructed Microbial Co-occurrence Networks

Across 90 soil samples collected from the North China Plain (Supplementary Figure 1), we obtained a total of 1,800,450 and 996,840 high-quality bacterial and fungal sequences, respectively, which were clustered into 65,761 bacterial operational taxonomic units (OTUs) and 4,033 fungal OTUs based on 97% sequence similarity. In this study, we selected OTUs that accounted for the top 20% in terms of relative abundance and occurring in more than 60% of all samples to construct co-occurrence networks (Jiao et al., 2019). In total, we identified 1,129 bacterial and 191 fungal dominant taxa (Supplementary Table 3), which occupied a small part of the individual total taxa (bacteria: 1.7%; fungi: 4.7%), but accounted for the majority of the total number of sequences, respectively (bacteria: 70.9%; fungi: 86.3%), which can maximize the representation of the whole community without data redundancy. The majority of bacterial sequences belonged to the phyla Proteobacteria (33.8%), Actinobacteria (21.6%), Acidobacteria (16.2%), Chloroflexi (7.2%), Gemmatimonadetes (6.3%) and Planctomycetes (3.9%). And the fungal sequences were mainly from the phyla Ascomycota (91.1%) and Basidiomycota (5.2%).

**FIGURE 1:**
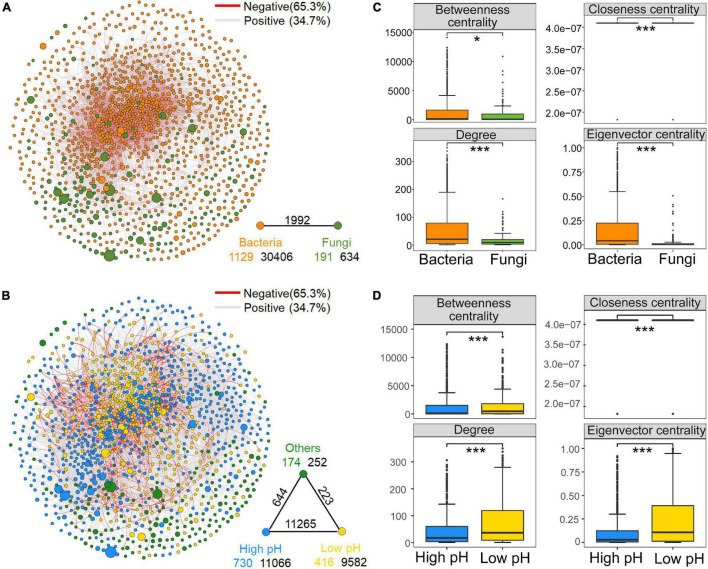
Meta-community co-occurrence network with nodes colored according to dominant microbial taxa [top: bacteria and fungi **(A)**] and ecological clusters [bottom: high pH, low pH, and others **(B)**] of soil microbiota on the North China Plain. The connection stands for a strong (Spearman’s *r* > 0.6) and significant (false discovery rate-corrected *P* < 0.01) correlation. The size of each node is proportional to the relative abundance of the operational taxonomic units (OTUs). A red edge indicates a negative correlation, and a gray edge indicates a positive correlation. A summary of node–edge statistics is provided to bottom right of the network. Colored numbers represent the number of nodes in corresponding categories; black numbers indicate the number of inner connections, and the numbers above edge connections represent the number of cross-community interactions. Node-level topological features for dominant microbial taxa **(C)** (top) and different ecological clusters **(D)** (bottom). *P < 0.05, **P < 0.01 and ***P < 0.001, based on Wilcoxon rank sum tests.

The constructed co-occurrence network consisted of 1,320 nodes (OTUs), including 1,129 bacterial OTUs and 191 fungal OTUs. A total of 33,032 edges (associations between OTUs) were inferred for the consensus microbial network (Figure 1A). Supplementary Figure 3 showed that the curves of network connectivity distribution fitted well with the power-law model, which was indicative of scale-free networks and significantly different from random networks. The associations were mainly observed among phylum Proteobacteria, Actinobacteria, Acidobacteria and Chloroflexi (Supplementary Figure 4 and Supplementary Table 4). The presence of positive edges (65.3%) is much greater than negative edges (34.7%) in the network. Bacterial nodes had the most internal connections, fewer edges between bacteria and fungi, and the fungal nodes had the fewest internal connections (Figure 1A). Moreover, values for the node-level topological features, including degree, eigenvector and closeness centrality were significantly higher (*P* < 0.001) in bacterial OTUs than in fungal OTUs (Figure 1C), which suggested that bacterial taxa played a more critical role in the network than fungal nodes.

**FIGURE 2:**
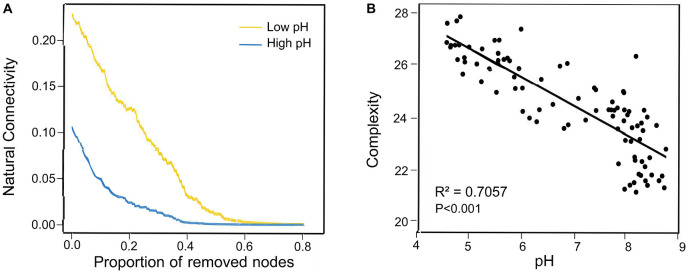
**(A)** Network robustness analysis of different ecological clusters (high pH and low pH). **(B)** Relationship between soil pH and co-occurrence network complexity (the ratio of edge to node).

Additionally, to explore the ecological preferences of soil microorganisms in the North China Plain, we divided the dominant bacterial and fungal taxa into two ecological clusters: high and low pH based on the Spearman’s correlations (*P* < 0.05) with soil pH, which had different habitat preferences (Supplementary Table 5). Co-occurrence network captured 730 nodes belonging to the high pH cluster and 416 nodes for low pH cluster, these taxa within the cluster that shared the same habitat and environmental preferences tended to co-occur (Figure 1B). Each of the ecological clusters consisted of multiple orders of soil microbiota. iii1–15, *Actinomycetales* and *Rhizobiales* tended to aggregate in soils with high pH, *Gaiellales*, *Solibacterales* and *Burkholderiales* prederred low soil pH, while *Xanthomonadales* and *Rhodospirillales* were relatively abundant in both high-pH and low-pH soils (Supplementary Table 5). Random forest analysis revealed that pH was the dominant environmental factor affecting the relative abundance of high and low pH clusters for bacterial and fungal communities (Supplementary Figure 5). Regarding the node-level topological features, the values of all parameters (including degree and betweenness, eigenvector and closeness centrality) in the low pH cluster were significantly higher than those in the high pH cluster (Figure 1D). Similar trends were seen in subnetworks constructed for bacterial community (Supplementary Figure 6A). These results showed that taxa of the low pH cluster, in comparison to those of the high pH cluster, were more often occupied the central positions in the network. Putative keystone species composition information below also confirms this conclusion (Supplementary Table 6). However, there was no significant difference in the node-level topological characteristics between high and low pH clusters in the fungal subnetwork (Supplementary Figure 6B). Moreover, when comparing the subnetwork stability of the high and low pH clusters, the natural connectivity at low pH was greater than that at high pH after removing the same proportion of nodes, which indicates the subnetwork of low pH cluster was more stable (Figure 2A).

### Co-occurrence Patterns of Dominant Bacterial and Fungal Taxa Driven by Environmental Filtering on the North China Plain

**FIGURE 3:**
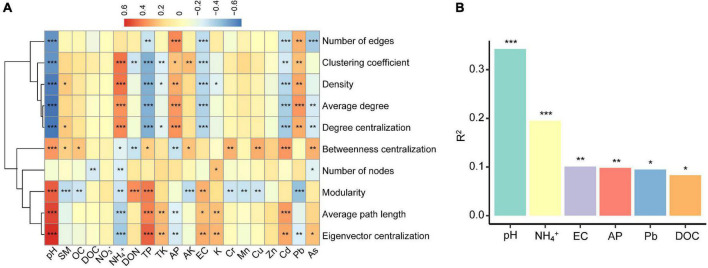
The importance of environmental factors for network-level topological features of meta-communities in individual **(A)** and aggregate **(B)** (Only the top six factors are shown to be significant). The R^2^ values was estimated with the multiple regression on distance matrices analysis, and asterisks represent significance of correlation (*P < 0.05, **P < 0.01, ***P < 0.001).

To examine the relative contribution of multiple environmental factors to the co-occurrence patterns on the North China Plain, we singled out 20 relatively independent (Spearman’s correlations: ρ^2^ < 0.6) environmental factors based on correlation (Supplementary Figure 2), and calculated a series of network-level topology characteristics for each soil sample. Based on correlation and hierarchical clustering analysis, the network-level topological features could be divided into two clusters (Figure 3A). The first cluster included number of edges, clustering coefficient, density, average degree, and degree centralization. The second cluster contained betweenness centralization, number of nodes, modularity, average path length, and eigenvector centralization. The first cluster of network-level topological features was positively correlated with soil pH, TP, EC, Cd, and As, and negatively correlated with NH_4_^+^, AP, and Pb, whereas the second clusters were on the contrary. Multiple regression on distance matrices (MRM; Figure 3B) showed that the contribution of pH (*R*^2^ = 34.2%, *P* < 0.001) to the network-level topological features overwhelmed than of other environmental factors. And the variance interpretation of each environment variable was shown in Table 1. The variation of each network-level topology characteristics could be well explained by the environmental variables. However, the explanatory quantities were not the same among the parameters, soil pH contributed the most toward explaining the variation in the topological features.

**TABLE 1:** Variation explained by environmental variables in the regression models for network-level topological features in wheat fields across the North China Plain.

Environmental Factors Network-level topological features	pH	NH_4_^+^	EC	AP	Pb	DOC	Total
Number of nodes		19.36%				4.58%	26.90%
Number of edges	25.98%		10.49%	9.91%	10.27%	3.74%	53.98%
Average degree	22.34%		13.42%	1.72%	5.48%		49.12%
Average path length	16.64%	12.89%					35.35%
Density		21.43%	2.40%	3.81%	5.62%		46.19%
Modularity	20.47%	10.59%	1.77%				49.75%
Clustering coefficient	28.7%	8.73%	3.93%	8.06%			54.19%
Degree centralization	17.91%			2.64%			42.12%
Betweenness centralization		4.50%		12.31%			28.01%
Eigenvector centralization	4.26%	20.91%	6.46%				39.07%

*NH_4_^+^, ammonium-nitrogen; EC, electrical conductivity; AP, available phosphorous; Pb, plumbum; DOC, dissolved organic carbon.*

**FIGURE 4:**
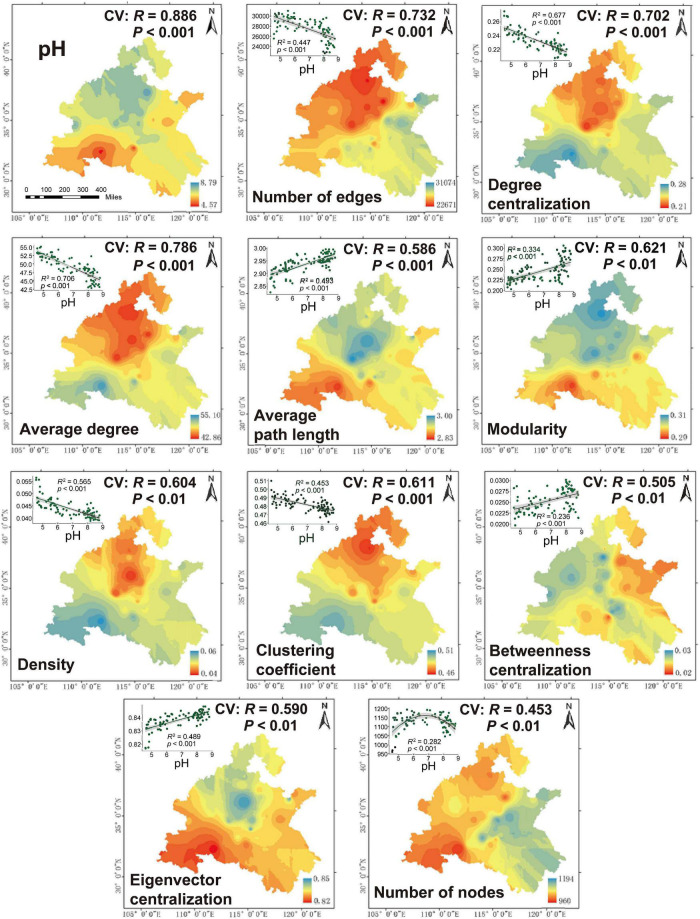
Spatial distribution of network-level topological features on the North China Plain. The cross-validation (“CV”) of the maps was calculated based on the Pearson correlation between the predicted and observed values at each sampling site. The relationship between network-level topological features and pH were estimated *via* linear least-squares regression analysis.

Furthermore, in order to better visualize the relationship between pH and network-level topological characteristics, we performed Kriging interpolation on pH and each network-level topological feature to obtain the spatial distribution maps (Figure 4). The predicted spatial patterns showed that the network-level topological features in the first cluster had higher values in the low pH regions than high pH regions. In contrast, the features in the second cluster were higher in high-pH soil than in low-pH soil. These observations were confirmed by multiple regression analysis. Significant and negative linear regressions were found between soil pH and the network-level topological features in the first cluster. However, the mostly features in the second cluster strongly increased with increasing pH, while the number of nodes peaked at neutral pH (Figure 4). Altogether, these results indicated that the ecological network of dominant bacterial and fungal taxa was more complex in low pH regions than that of high pH regions. In order to avoid the effect of microbial abundance to the ecological network when illustrate the change of the network pattern along pH gradient, the ratio of links to nodes were calculated as the network complexity. The result of Pearson correlation between network complexity and pH reconfirmed that the network of dominant bacterial and fungal taxa was significantly affected by pH, with an R^2^ of 0.7057 (*P* < 0.001) and was more complex in low pH than that of high pH (Figure 2B). For the sub-network generated for bacteria, the same network-level topological characteristics and pH variation patterns were observed, but not for fungal subnetworks (Supplementary Figures 7, 8).

**FIGURE 5:**
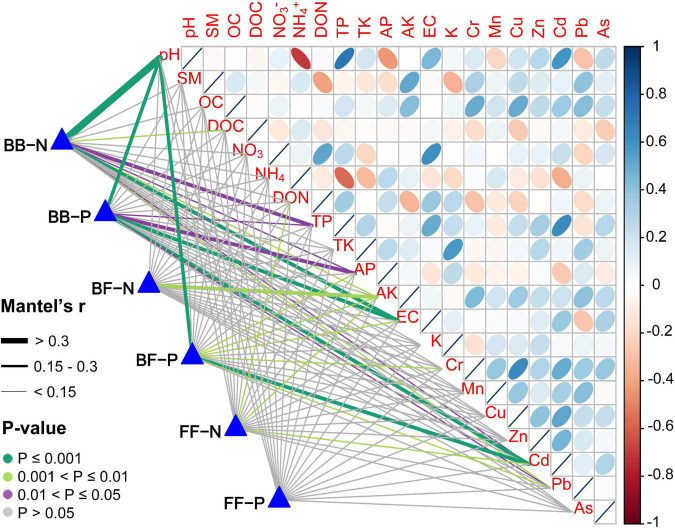
Correlation of environmental factors with inter-community and intra-community associations. Pairwise comparisons of environmental factors are shown, with a color gradient denoting Spearman’s correlation coefficient. The number of positive and negative associations within and between communities were related to each environmental factor by Mantel tests. Edge width corresponded to the Mantel’s r statistic for the corresponding distance correlations, and edge color denoted the statistical significance based on 9,999 permutations. BB included associations only between bacteria-bacteria; BF included associations only between bacteria-fungi; FF included associations only between fungi-fungi. P: positive; N: negative.

In addition, to determine how the number of connections between subnetworks changed, we applied regression analysis and found there were significantly negative relationships between soil pH and the number of positive/negative edges within the bacterial community. Moreover, the number of positive edges between the two kingdoms decreased with increasing pH, whereas the number of negative edges peaked at neutral pH. For the fungal community, there was no significant correlation between the number of positive and negative edges and pH (Supplementary Figure 9). The links between the number of positive and negative associations within and between communities and each environmental factor were detected by Mantel tests (Figure 5). Number of connections in the bacterial network were significantly correlated with pH, TP, AP, EC, and Cd. The number of positive edges between kingdoms was significantly affected by pH and Cd, and AK was correlated to the number of negative edges between kingdoms. However, no significant relationships among fungal community connections number and soil factors were observed.

### Linkage Among Keystone Taxa and Soil Properties

**FIGURE 6:**
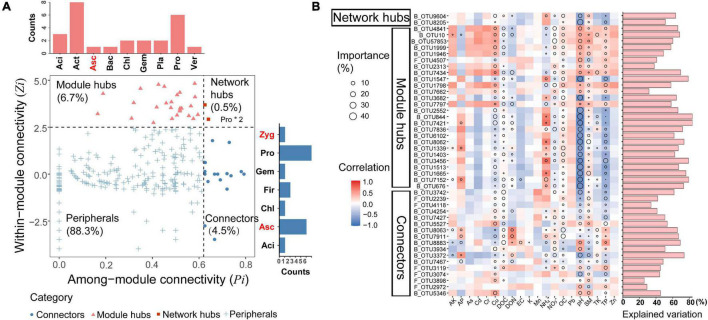
Identification of potential keystone taxa and their relative abundance in relation to environmental factors. **(A)** Z-P plot showing the classification of nodes to identify putative keystone taxa of ecological network. Each symbol represents an OUT. The bar charts on the top and right represent critical phylum in module hubs and connectors, respectively. Black represents the phylum of bacteria and red represents the phylum of fungi. Network hubs composition in the box. Aci, Acidobacteria; Act, Actinobacteria; Asc, Ascomycota; Bac, Bacteroidetes; Chl, Chloroflexi; Fir, Firmicutes; Gem, Gemmatimonadetes; Pla, Planctomycetes; Pro, Proteobacteria; Ver, Verrucomicrobia; Zyg, Zygomycota. **(B)** Environmental contributions to the distributions of keystone taxa in wheat fields across the North China Plain. Correlation and multiple regression model for the relative abundance of keystone taxa and environmental factors were shown in heatmap. Circle size represents the variable importance (i.e., the proportion of explained variance calculated *via* multiple regression modeling and variance decomposition analysis). Colors represent Spearman correlations.

Keystone nodes (network hubs, module hubs, and connectors) were identified by analyzing the topological roles that each node played in the network of dominant bacterial and fungal taxa (Figure 6A). In this study, a total of 2 network hubs, 26 module hubs, and 17 connectors were detected in the microbial network. Both network hubs belonged to the phylum Proteobacteria. The detected module hubs were composed of six taxa within the Proteobacteria, five taxa within Ascomycota, two taxa within Firmicutes, and one taxon within each of the phyla Acidobacteria, Chloroflexi, Gemmatimonadetes and Zygomycota. The connectors consisted of eight taxa within the Actinobacteria, six taxa within Proteobacteria, three taxa within Acidobacteria, two taxa within Planctomycetes, Gemmatimonadetes, Chloroflexi, one taxon within Verrucomicrobia, Ascomycota, and Bacteroidates (Supplementary Table 6). Additionally, significant and negative linear correlations were found between pH and the relative abundance of keystone taxa (Supplementary Figure 10), suggesting that increasing soil pH reduced the number of keystone taxa significantly correlated with network complexity.

To explore the linkages between environmental parameters and the relative abundance of keystone taxa, we then correlated the keystone taxa to soil properties and identified the major drivers for each keystone taxon (Figure 6B). Spearman correlation analysis revealed that there were distinct environmental preferences between keystone taxa. Compared with network hubs and module hubs, connectors had less correlation with environmental variables, suggesting that they were more resistant to environmental changes. Moreover, the results of multiple regression model showed that pH was the most important predictor for the relative abundance of keystone taxa, TP, SM, OC, Cu, DOC, and NO_3_^–^ also played important roles.

## Discussion

The sheer complexity of microbial communities makes it challenging to assess potential microbial associations (van Dijk et al., 2014). Therefore, analysis of co-occurrence networks has been widely used to infer potential associations between microorganisms, and for the most part seems to only way forward since experimental culturing or removal experiments are prohibitively difficult and time consuming, and limited in the numbers of interactions they can study simultaneously (Faust et al., 2012; Jiang et al., 2017; Fan et al., 2018). Here, we have constructed co-occurrence networks of bacteria and fungi in soil from wheat fields across the North China Plain as an example of an important agricultural region. We explored the network topological features and keystone taxa geographic patterns, and demonstrated that different microbiomes and different ecological clusters differed in terms of network topological features showing strong correlations with certain environmental variables. The results provide a basis for further understanding the distribution pattern and influencing factors of microorganisms in agricultural ecosystem.

### Soil pH Dominates the Geographic Patterns of Microbial Network Topological Features Across the North China Plain

The role of environmental pH shifts is a prominent topic in the study of interspecies interactions. Following on from the study by Shi et al. (2021) which explored high and low pH as discontinuous and discrete categories, we found that in fact the topological characteristics of the co-occurrence network influenced by soil pH vary along a continuous gradient, and that the influence of pH was far greater than any other measured environmental factors. This is broadly consistent with recent studies of soybean fields showing that soil pH plays a key role in rhizosphere microbial interactions (Zhang et al., 2018).

Soil pH shapes microbial metabolism in different ways. Firstly, as an integrating index to measure soil conditions (Lauber et al., 2009), soil pH is highly correlated with a wide range of biogeochemical conditions [e.g., NH_4_^+^, TP, AP and many metal ions (Figure 4)], which significantly affect the environmental conditions related to microbial growth and survival (Pan et al., 2014; Ma et al., 2018). The pH also affects the activity of extracellular enzymes and the reactivity of natural organic matter (Paul et al., 2006). Secondly, soil pH directly imposes a physiological constraint on soil microorganisms (e.g., in affecting homeostasis of intracellular pH), and any slight change in pH value unit will have a significant impact on microbial growth and metabolic activity (Fernandez-Calvino and Baath, 2010). Additionally, pH may also affect the rate of energy expenditure in microbial respiration and hence microbial community structures by regulating the thermodynamics and kinetics of redox reactions (Jin and Kirk, 2018). Therefore, it is reasonable that one would find a close connection between pH and microbial interactions at the community level.

In general, taxa of microorganisms (especially prokaryotes) show preferences for ranges of soil pH, with the greatest species diversity and abundance in neutral soils, “acidity specialists” exist in acidic soils as well as “alkalinity specialists” in alkaline soils (Barberán et al., 2012; Jones and Bennett, 2017; Vieira et al., 2020). Among the low-pH cluster of microbial taxa that we detected, the abundance of *Xanthomonadales* has been found to be significantly negatively correlated with pH (Zhang et al., 2020), while *Nocardioidaceae* and *Sordariales* from the high-pH cluster, favors high-pH environments (Huang et al., 2017). In addition, we found a unique bacterial class *Gammaproteobacteria*, which proved to be widespread in wheat fields with low pH and high NO_3_^–^-N concentrations (Hamamoto et al., 2018). The main families within this class were *Chromatiaceae*, *Sinobacteraceae*, and *Xanthomonadaceae*, all of which have been reported as only being found in agricultural soils (Kuramae et al., 2012), which could be used as indicator groups in farmland ecosystems.

### Patterns of Microbial Community Interactions Along the pH Gradient

Co-occurrence networks represent strong correlations between OTUs, indicating potential species-species interaction in communities (Ma et al., 2018). In the present study, we found that bacteria and fungi were more closely related within their respective community, suggesting a wide range of interactions between species such as the exchange of metabolites (Woyke et al., 2006) and cooperation in biofilm construction (Rodríguez-Martínez and Pascual, 2006). We also observed some correlations between microbes in the two kingdoms. It is no surprise that several bacterial taxa, such as *Burkholderia*, *Sphingomonas* and *Pseudomonas*, have been found to interact with fungi in soil (Gamalero et al., 2008; Warmink and van Elsas, 2008). In general, the products (such as water-soluble sugars and phenolic compounds) released by fungi during the degradation of refractory organic matter (lignin and cellulose) in soil can be utilized by bacteria (Boer et al., 2005). Although fungi can produce a portion of carbon, plant root exudates (i.e., amino acids, sugars and organic acids) are considered to be the main carbon source for bacteria (Philippot et al., 2013). Therefore, the nutrient dependence of bacteria on fungi in farmland ecosystems may be low, resulting in a low proportion of bacteria-fungus edges in the co-occurrence network. In addition, most of the network links were between bacteria: often linking Proteobacteria, Actinobacteria and Acidobacteria with other bacterial phyla. Amongst the fungi, there were many positive associations among Ascomycota, which is consistent with previous results, that is, phylogenetically related taxa are also ecologically related (Barberán et al., 2012). This suggests that these ascomycetes may either have synergies or share similar ecological niches in the farmland environments.

In this study, we observed that the number of nodes in the co-occurrence network peaked at about pH 7, which is consistent with previous studies confirming the highest diversity of soil bacterial communities with near-neutral pHs (Lauber et al., 2009). However, microbial taxa were more closely correlated in the low pH area than in the high pH area, this may be due to the close contact between Acidobacteria and other microbial groups (Supplementary Table 4), which has been shown to increase toward lower pH (Jones et al., 2009). In addition, soil acidity supports basic soil properties and functions, such as the solubility of exchangeable ions and nutrients, or the use of internal and external acid gradients by bacteria to produce ATP and rotate flagella motors (Braus and Whitman, 2021). As far as is known, the large pH gradient between the inside and outside of the cell causes microbes living in an alkaline environment to greatly reduce the proton dynamics (PMF) used to produce ATP—much less so than for acidophiles (REF). This makes it energetically less favorable for alkaliphiles to generate energy through oxidative respiratory chains and oxidative phosphorylation (Padan et al., 2005), and results in a decrease in the frequency of microbial interaction in the community.

Understanding ecological niches is important for determining the mechanisms of community assembly (Jiao and Lu, 2020). Here, we further investigated the differences of environmental adaptive capabilities between different ecological clusters (high pH and low pH). We found that the niche breadth of low pH cluster was narrower than that of high pH cluster (Supplementary Figure 11), implying that interspecific interactions (such as competition, etc.) are stronger in low pH clusters, thus the co-occurrence network is more closely connected. Deterministic processes tend to have a stronger impact on the habitat specialists with a narrow niche breadth than on generalists with a wide niche breadth (Pandit et al., 2009). Therefore, it is perhaps unsurprising that the lower pH regions have more stable co-occurrence network structures. However, this observation is inconsistent with previous studies on the Tibetan Plateau, and we reason that this difference might be mainly since Chen et al. (2021) explored the associations of microbial communities in the pH range under the influence of different vegetation types (alpine steppe and alpine meadow) and did not study a unified microhabitat.

It is also interesting that in our NCP study, the pH-related trend is essentially dominated by bacteria. Fungi by themselves showed no significant trend, while bacteria alone—and fungi in combination with bacteria—showed the same clear trend. Generally, fungal community structure shows much weaker trends in relation to pH than bacteria, and fungi generally exhibit wider pH ranges for optimal growth. For instance, the fungal genus *Mariannaea*, has been found to grow between pH 5.5–8.5 in culture-based studies (Domsch et al., 1980), which may relate to the larger cells and much lower surface to volume ratio of fungi, which enables better cellular pH homeostasis (Beales, 2004).

Unlike the trend in network connectivity, the associations within communities were most stable at neutral pH (Supplementary Figure 9), where they were dominated by negative associations. This greater stability may reflect the greater available energy and resources for niche specialization by bacterial cells at around neutral pH, since high and low pH are each thought to act as a drain on resources due to the requirements for cellular homoeostasis, relative to neutral pH (Lauber et al., 2009). The high ratio of negative interactions may reflect narrow specialized niches and strong competitive exclusion at around neutral pH.

### Linkages Between Keystone Taxa and Ecosystem Functions

Through network analysis, keystone taxa can often be detected as network hubs, module hubs, and connectors (Olesen et al., 2007; Faust and Raes, 2012). Only a few hubs or connectors have a wide distribution across different plots, supporting the environmental dependency theory that keystone taxa do not dominate anywhere or at any time, but only play a key role in specific environments (Abdul Salam et al., 2013). We noted that the relative abundance of keystone taxa decreased as soil pH increased, consistent with the previous observation that the putative keystone taxa change with conditions (Lu et al., 2013). Although they were not abundant, these low abundance keystone taxa may have disproportionately large impacts on ecosystem functions and services (Lynch and Neufeld, 2015). Such a network hub, *Oxalobacteraceae*, has been previously found to be closely related to plant growth and nitrogen acquisition (Yu et al., 2021). Module hub *Burkholderiales* and connector *Rhizobiles*, are dominant members of the rhizosphere microbiome and are considered keystone taxa across different ecosystems (Banerjee et al., 2018). Another connector, *Mortierella*, has the ability to decompose complex organic substrates and with an important phosphate lysozyme that promotes soil activity and plant growth (Zhang et al., 2011). In addition, taxa within the phyla Gemmatimonadetes and Planctomycetes are often associated with the rhizosphere of plants and indirectly promote plant growth by participating in a variety of ecological processes, which have also been identified as kinless hubs in previous studies (Shi et al., 2020). The presence of these keystone taxa could thus be critical to maintaining soil health and crop productivity. Hence, manipulation of keystone taxa in microbial network structures, such as the addition or removal of functionally specific keystone taxa, may provide a promising approach for agricultural management to improve crop yields (Banerjee et al., 2018).

## Conclusion

In this study, network analysis was used to integrate the complex associations between bacterial and fungal microbiota in the soil of wheat fields across the North China Plain, into the predictable topological features of the co-occurrence network, providing valuable insights for the study of microbial communities in farmland soil at large spatial scales. Our study identified that soil pH plays a key role in driving the unique geographical pattern of the co-occurrence network topology of soil microbial communities, and microbial taxa are more closely related in the low pH region than in the high pH region. The relative abundance of keystone taxa that are important for ecosystem function and stability is also strongly affected by pH. These findings suggest a trend toward more integrated and specialized community functioning at lower pH. Analyzing and understanding the basis of these trends may ultimately contribute to a better understanding of the geographic patterns of soil microbial interactions in farmland ecosystems, and may provide microbiologists and agronomists with more targeted indicators to monitor and ultimately improve soil health.

## Data Availability Statement

The original contributions presented in the study are included in the article/Supplementary Material, further inquiries can be directed to the corresponding author/s.

## Author Contributions

YY: methodology, formal analysis, visualization, and writing—original draft. YS: methodology, validation, supervision, and writing—review and editing. JF: data curation and investigation. HC and JA: conceptualization, supervision, and writing—review and editing. All authors contributed to the article and approved the submitted version.

## Conflict of Interest

The authors declare that the research was conducted in the absence of any commercial or financial relationships that could be construed as a potential conflict of interest.

## Publisher’s Note

All claims expressed in this article are solely those of the authors and do not necessarily represent those of their affiliated organizations, or those of the publisher, the editors and the reviewers. Any product that may be evaluated in this article, or claim that may be made by its manufacturer, is not guaranteed or endorsed by the publisher.

## Footnotes

1
http://www.resdc.cn/

2
http://qiime.sourceforge.net/

3
http://greengenes.secondgenome.com/

4
http://gephi.github.io/

5
http://www.esri.com/software/arcgis/arcgis-fordesktop
