# Supplementary material for: Soil Microbial Network Complexity Varies With pH as a Continuum, Not a Threshold, Across the North China Plain
Source: Front Microbiol. 2022 Jun 6;13:895687. doi: 10.3389/fmicb.2022.895687 (PMC9207804; doi:10.3389/fmicb.2022.895687)
Supplement: Supplementary file 2 [file Table_1.DOCX]

Soil microbial network complexity varies with pH as a continuum, not a threshold, across the North China Plain

**Supplementary material**


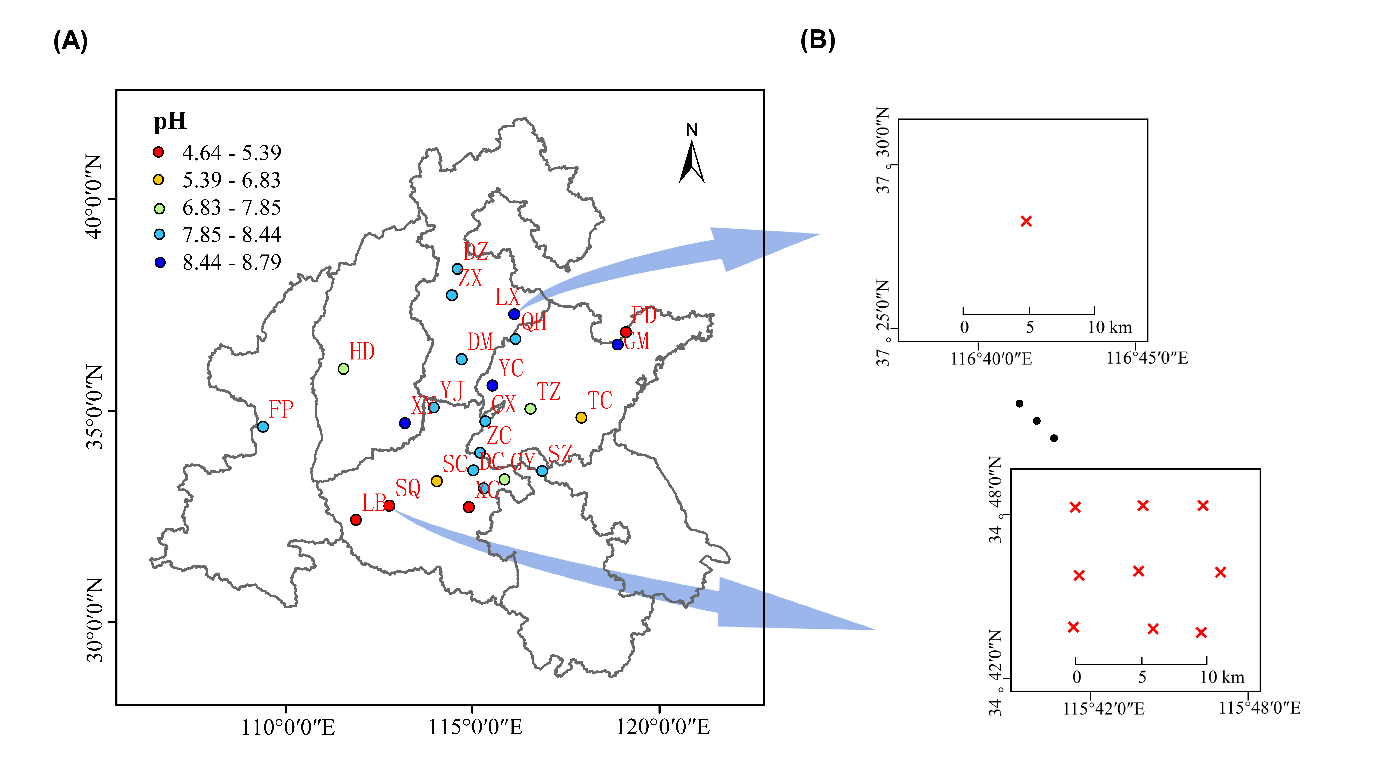


Fig S1. Geographical location of the sampling sites (A) and quadrat sets (B) in wheat field of the North China Plain. Colored by pH (n=90). Sample sites are colored according to pH value, and each site has a different number of plots.


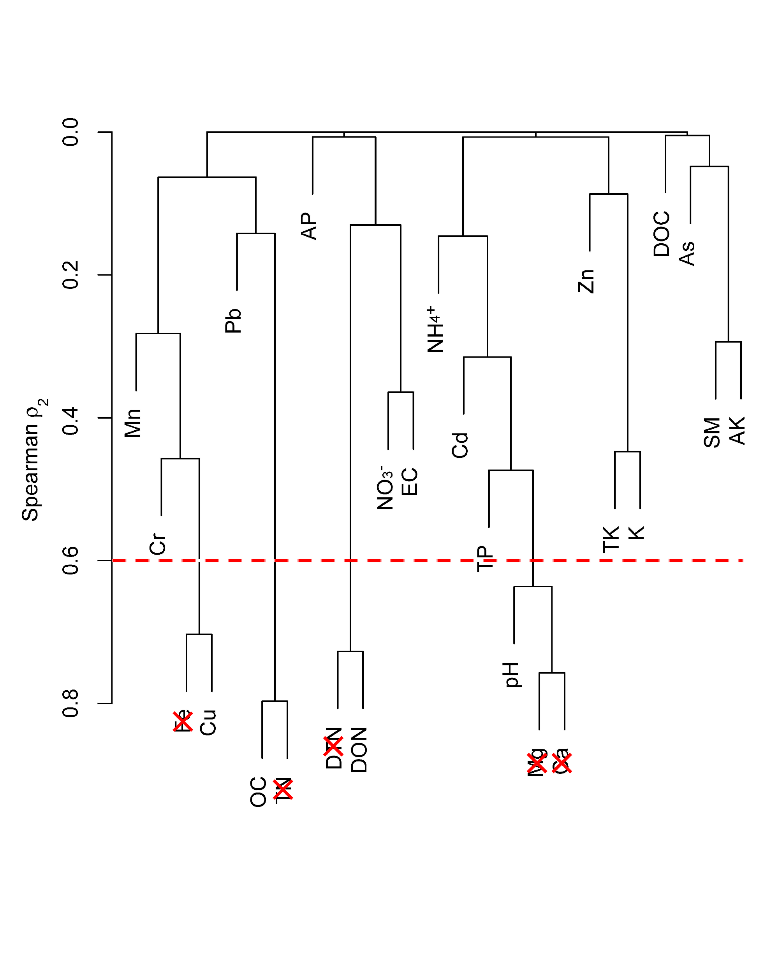


Fig S2. Cluster analysis of the measured environmental variables in maize and rice fields. If the Spearman correlation between environmental factors is greater than 0.6, only one parameter is retained. The analysis was performed and plotted using “varclus” in “Hmisc” R package.





Fig S3. Co-occurrence network connectivity distribution of microbial communities


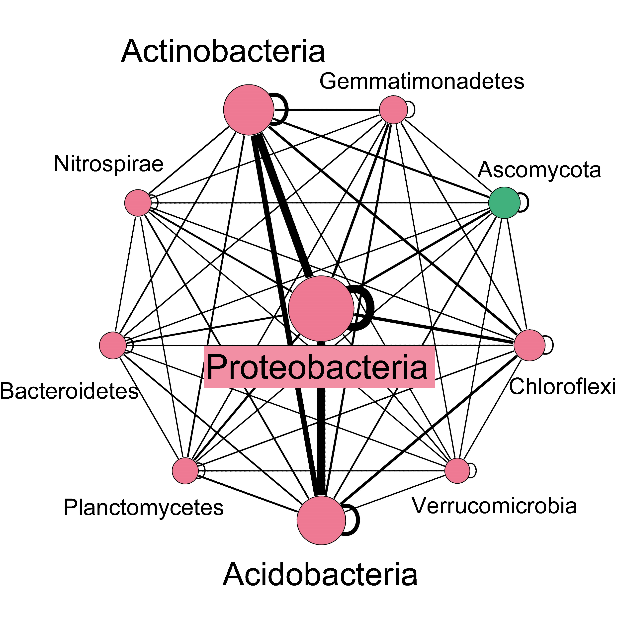


Fig S4. Microbial co-occurrence network on the North China Plain based on phylum level. The nodes represent phylum, red for bacteria and green for fungi. The thickness of lines matches the number of edges


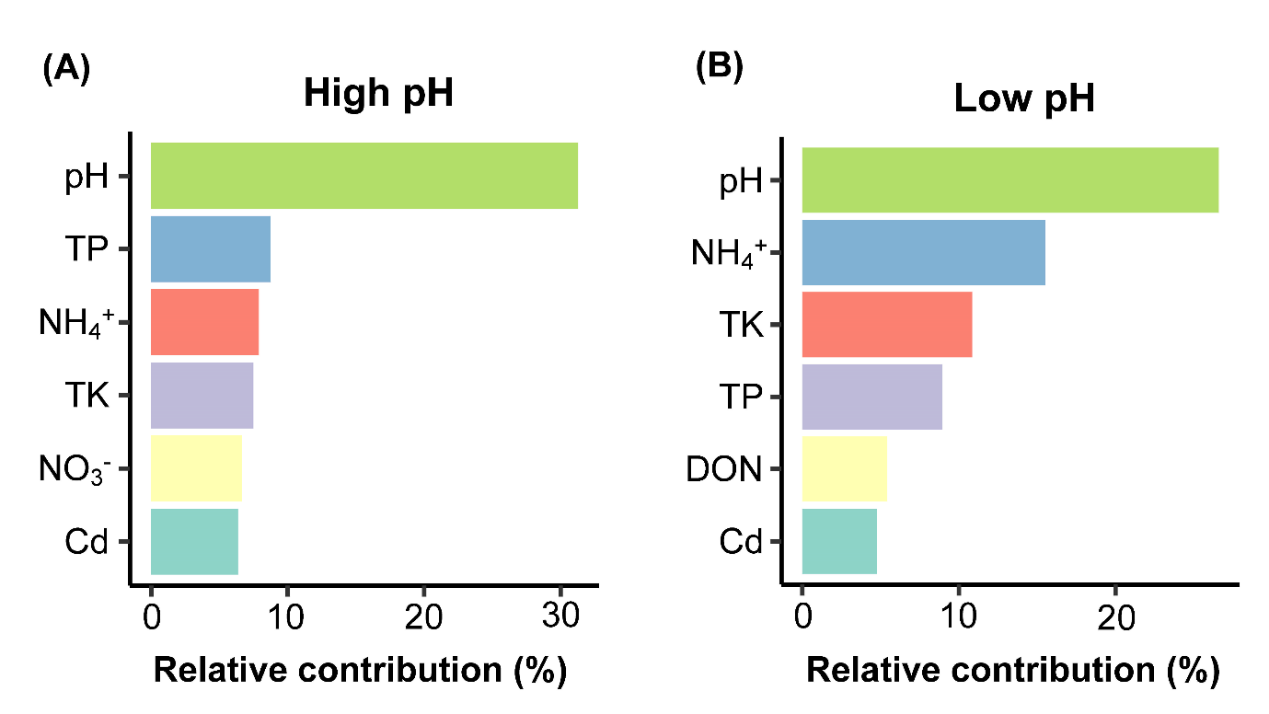


Fig S5. Random forest analysis to explain the variation caused by environmental factors in the relative abundance of dominant taxa in high-pH (A) and low-pH (B) cluster for soil microbiota on the North China Plain.


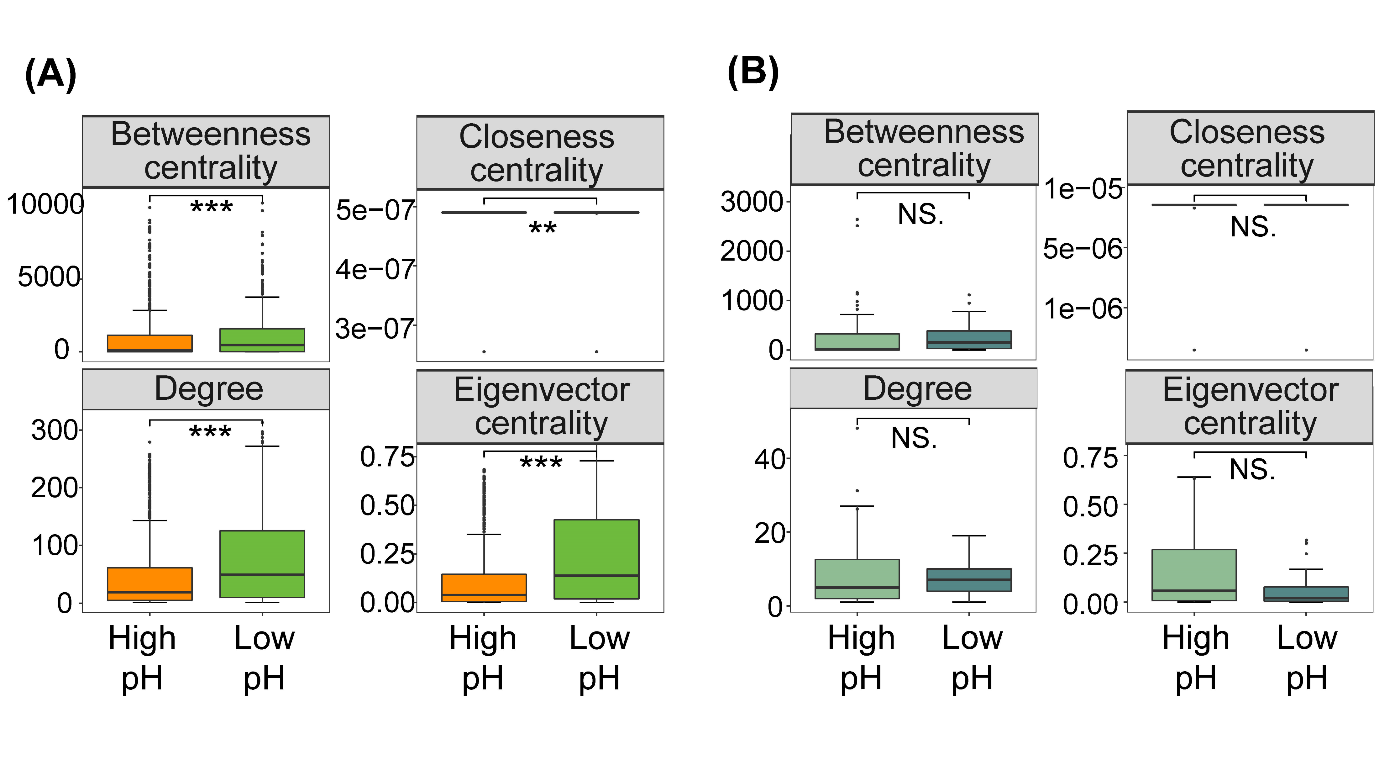


Fig S6. Unique node-level topological features of the dominant bacterial (A) and fungal (B) taxa assigned to high- and low-pH clusters in the North China Plain. The degree, betweenness, closeness, and eigenvector centrality are shown. NS: P>0.05, ^*^*P* < 0.05, ^**^*P* < 0.01, and ^***^*P* < 0.001 based on Wilcoxon rank sum tests.


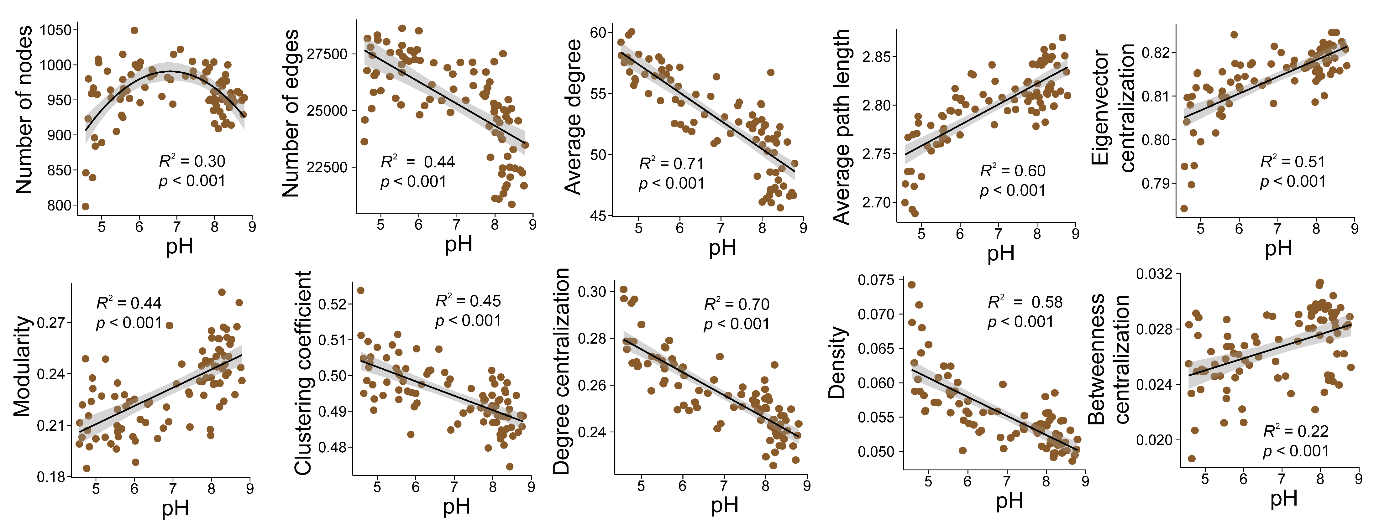


Fig S7. Relationship between pH and network-level topological properties of bacterial communities.


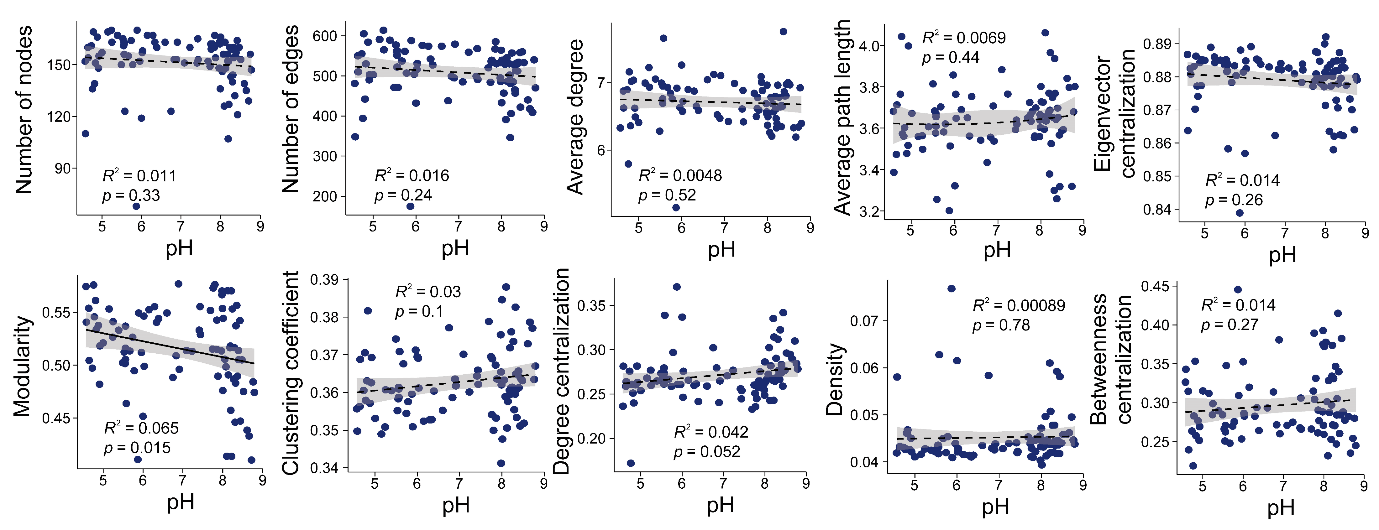


Fig S8. Relationship between pH and network-level topological properties of fungal communities.

**
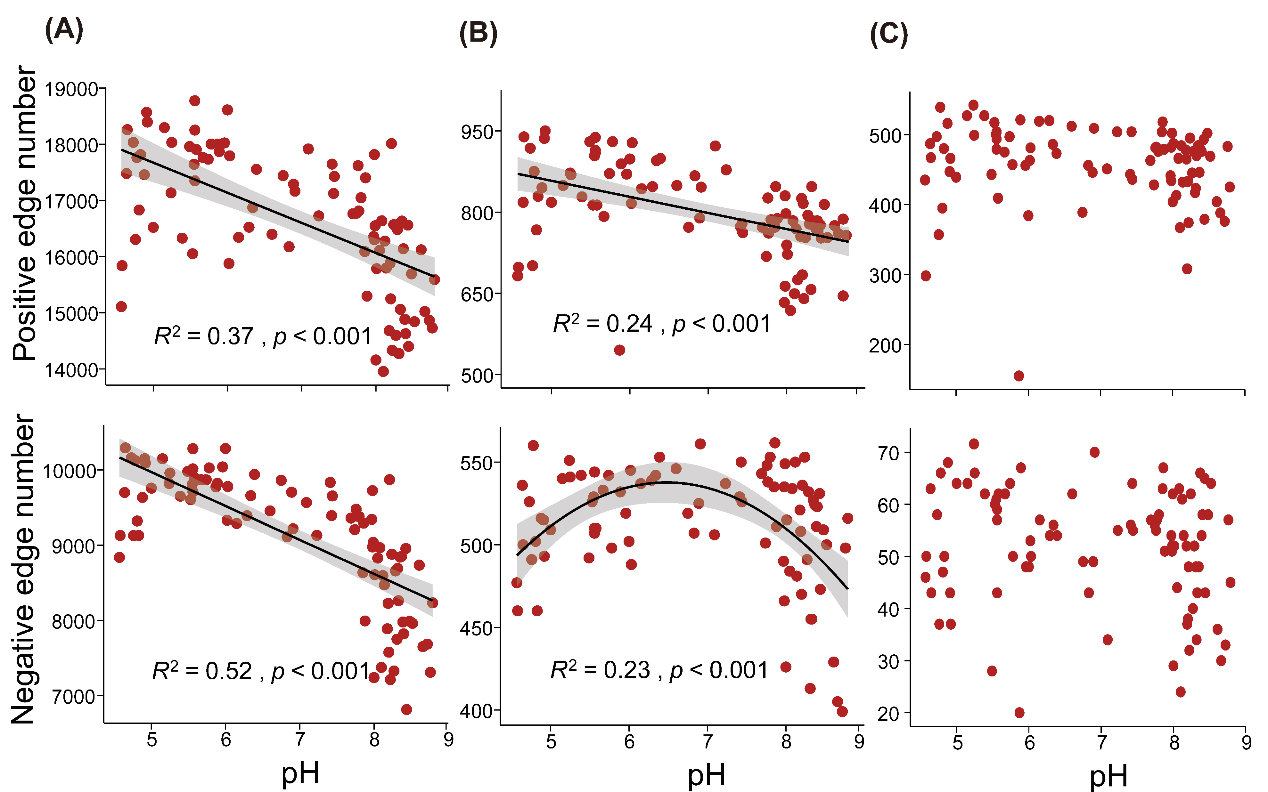
**

Fig S9. The number of positive and negative associations within and between the combined communities (bacteria and fungi) varied with soil pH. (A)BB; (B)BF; (C)FF. BB included associations only between bacteria-bacteria; BF included associations only between bacteria-fungi; FF included associations only between fungi-fungi.


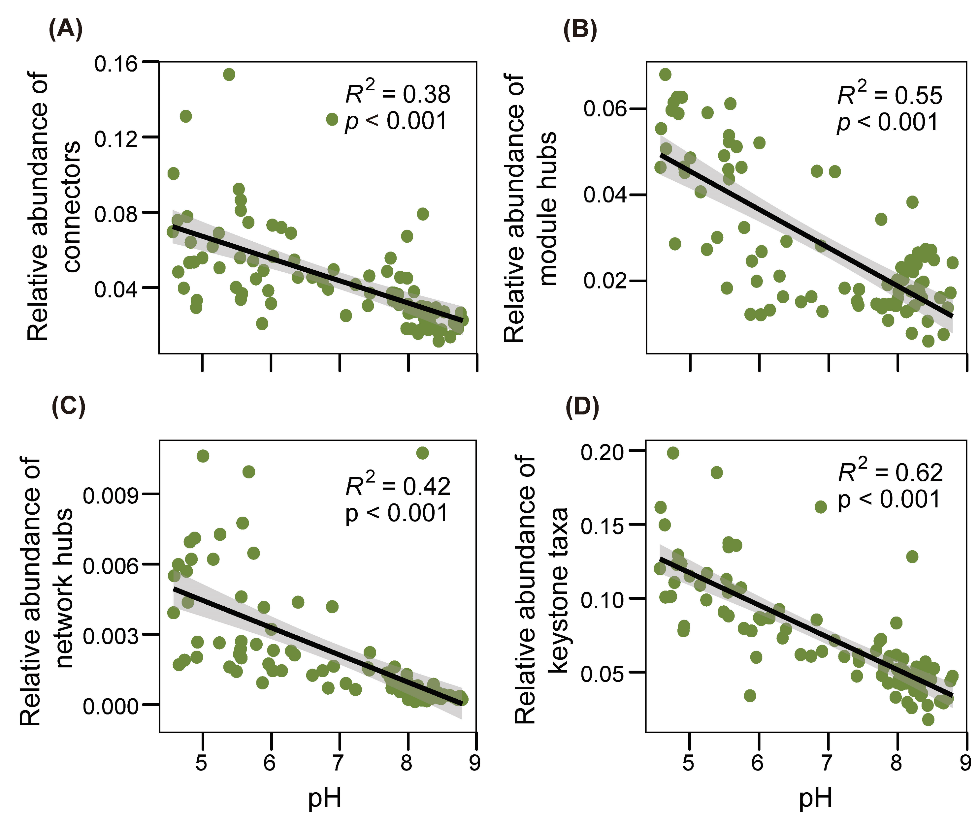


Fig S10. Correlation between soil pH and relative abundance of different keystone taxa of meta-community (bacteria and fungi). (A)Connector; (B)Module hub; (C)Network hub; (D) Total.


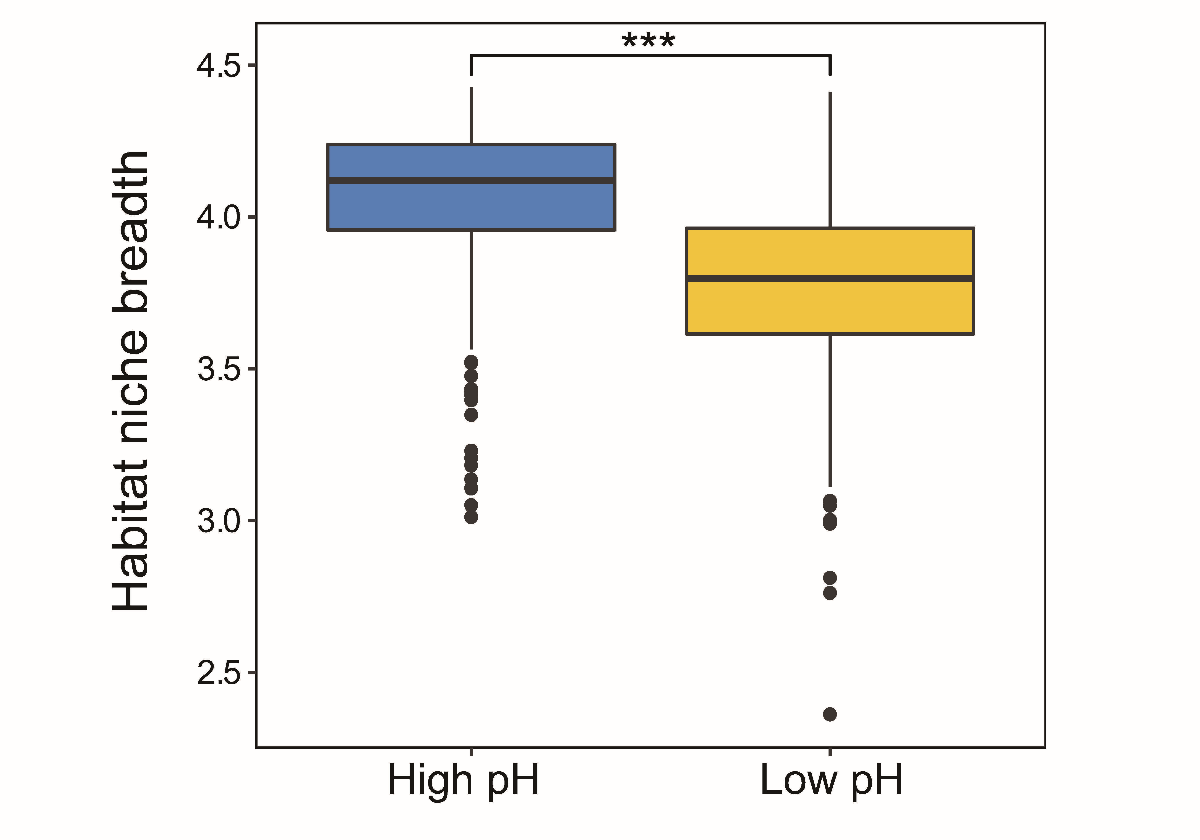


Fig S11. Comparison of mean habitat niche breadth for high and low pH clusters (asterisks indicate significant difference using T.test, *** means p<0.001).
