# Supplementary material for: Soil Microbial Network Complexity Varies With pH as a Continuum, Not a Threshold, Across the North China Plain
Source: Front Microbiol. 2022 Jun 6;13:895687. doi: 10.3389/fmicb.2022.895687 (PMC9207804; doi:10.3389/fmicb.2022.895687)
Supplement: Supplementary file 4 [file Table_3.docx]

**Table S2.** Description of node- and network-level topological features in the co-occurrence network for soil microbiota


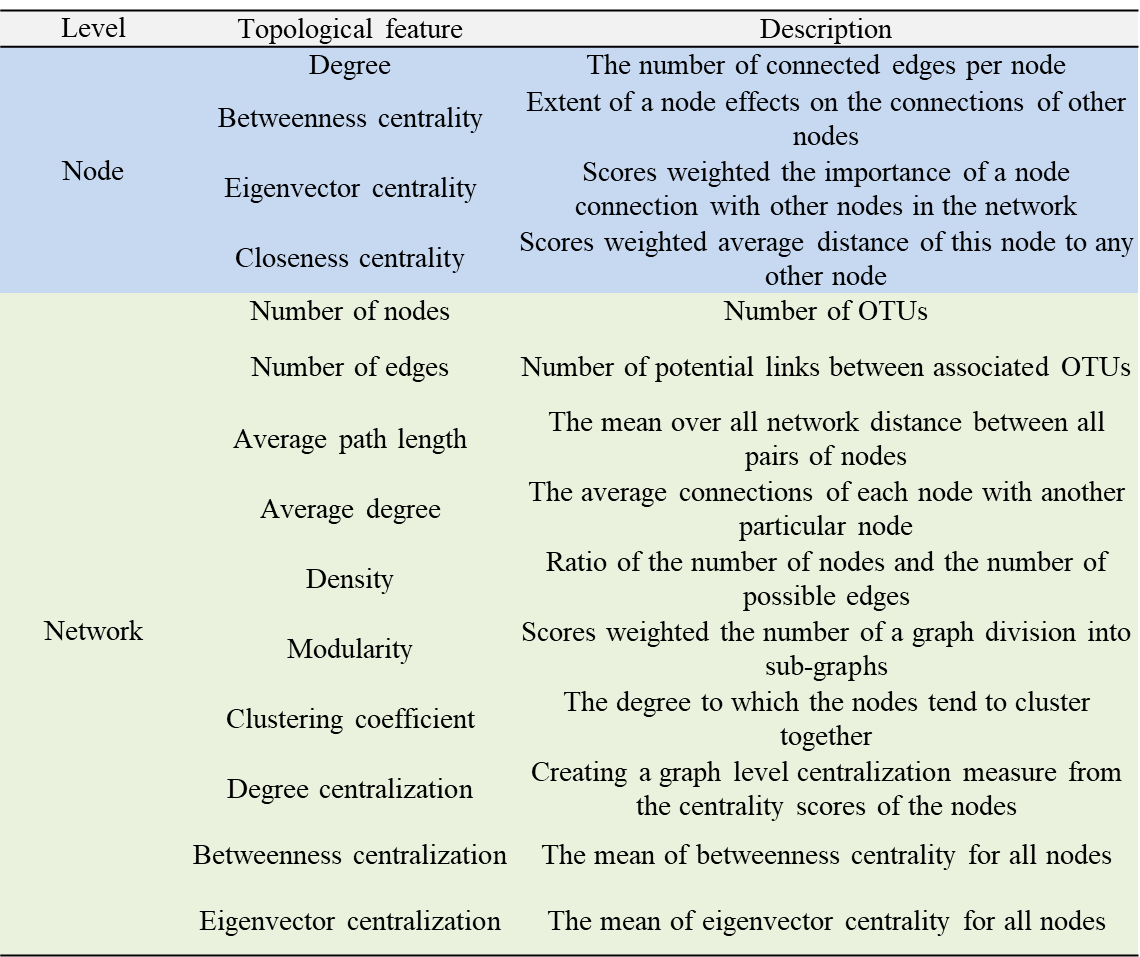


Reference: Ma, B., H. Wang, M. Dsouza, J. Lou, Y. He, Z. Dai, P. C. Brookes, J. Xu, and J. A. Gilbert. 2016. Geographic patterns of co-occurrence network topological features for soil microbiota at continental scale in eastern China. ISME J 10 (8):1891-1901.
