# Supplementary material for: Soil Microbial Network Complexity Varies With pH as a Continuum, Not a Threshold, Across the North China Plain
Source: Front Microbiol. 2022 Jun 6;13:895687. doi: 10.3389/fmicb.2022.895687 (PMC9207804; doi:10.3389/fmicb.2022.895687)
Supplement: Supplementary file 6 [file Table_5.docx]

Table S4. The number of connections between taxa (phylum level) in the microbial network.

| **P**  **N** | **P1** | **P2** | **P3** | **P4** | **P5** | **P6** | **P7** | **P8** | **P9** | **P10** |
| --- | --- | --- | --- | --- | --- | --- | --- | --- | --- | --- |
| **P1** | 1268/573 | 623/611 | 25/12 | 108/40 | 307/201 | 216/143 | 139/34 | 233/129 | 1179/1062 | 22/4 |
| **P2** | 974/577 | 1565/236 | 142/55 | 31/95 | 410/121 | 186/120 | 83/34 | 176/108 | 1731/753 | 9/7 |
| **P3** | 126/90 | 278/75 | 510/71 | 23/9 | 43/20 | 36/12 | 7/8 | 32/6 | 430/169 | 3/2 |
| **P4** | 75/42 | 29/69 | 6/4 | 92/0 | 10/20 | 34/7 | 2/6 | 23/13 | 74/80 | 16/0 |
| **P5** | 288/165 | 255/90 | 10/5 | 8/17 | 106/40 | 48/30 | 28/8 | 68/25 | 366/258 | 5/1 |
| **P6** | 170/65 | 84/61 | 6/0 | 31/2 | 48/26 | 92/11 | 15/4 | 62/9 | 158/97 | 13/1 |
| **P7** | 113/27 | 34/39 | 6/1 | 0/1 | 18/14 | 11/11 | 29/0 | 16/5 | 94/74 | 0/1 |
| **P8** | 50/23 | 48/7 | 11/0 | 12/0 | 17/6 | 37/4 | 1/0 | 43/3 | 75/13 | 9/0 |
| **P9** | 1464/1017 | 1301/854 | 95/46 | 211/63 | 444/288 | 393/186 | 158/88 | 318/209 | 2827/1769 | 60/6 |
| **P10** | 7/0 | 4/1 | 6/0 | 6/0 | 1/0 | 4/0 | 0/2 | 6/0 | 4/1 | 5/0 |

Abbreviation: P1: *Acidobacteria*; P2: *Actinobacteria*; P3: *Ascomycota*; P4: *Bacteroidetes*; P5: *Chloroflexi*; P6: *Gemmatimonadetes*; P7: *Nitrospirae*; P8: *Planctomycetes*; P9: *Proteobacteria*; P10: *Verrucomicrobia*.
